# Research Progress About Glioma Stem Cells in the Immune Microenvironment of Glioma

**DOI:** 10.3389/fphar.2021.750857

**Published:** 2021-09-23

**Authors:** Xiangyu Li, Ming Liu, Junfeng Zhao, Tong Ren, Xin Yan, Lijun Zhang, Xun Wang

**Affiliations:** ^1^ Department of Neurosurgery, The Third People’s Hospital of Dalian, Non-Directly Affiliated Hospital of Dalian Medical University, Dalian, China; ^2^ Department of Neurosurgery, Ningde Municipal Hospital Affiliated of Ningde Normal College, Ningde, China; ^3^ Department of Medical Oncology, The Third People’s Hospital of Dalian, Non-Directly Affiliated Hospital of Dalian Medical University, Dalian, China; ^4^ Department of Ophthalmology, The Third People’s Hospital of Dalian, Non-Directly Affiliated Hospital of Dalian Medical University, Dalian, China

**Keywords:** glioma, glioma stem cells, microenvironment, immune suppression, treatment

## Abstract

Gliomas are the most common primary tumors of the central nervous system. Due to the existence of the blood-brain barrier and its unique regional immune characteristics, the study of the immune microenvironment of gliomas is particularly important. Glioma stem cells are an important cause of initiating glioma, promoting tumor progression and leading to tumor recurrence. Immunotherapeutic strategies targeting glioma stem cells have become the focus of current research. This paper will focus on the research progress of glioma stem cells in the immune microenvironment of glioma to provide the basis for the immunotherapy of glioma.

## Introduction

Glioma is one of the most common malignant tumors of the central nervous system. According to the statistics of brain Tumor Registry in the United States, glioma accounts for about 80% of all malignant tumors of the central nervous system, and more than half of gliomas are glioblastoma (GBM) (Ostrom et al., 2015). GBM growth is aggressive, causes tumor recurrence, migration, so that it is difficult to cure. Currently, the standardized treatment plan for glioma, namely, the maximum safe surgical resection of the tumor supplemented by postoperative concurrent chemoradiotherapy or adjuvant chemotherapy, has a very poor prognosis with a median survival of only 15 months due to its rapid growth, high invasiity and multi-tolerance of chemoradiotherapy (Eder and Kalman, 2014; Wang et al., 2016). Even emerging molecular targeted therapy, electric field therapy and other methods, but these can only alleviate the disease to a limited extent, there is no significant improvement in overall survival. Recent studies have shown that the brain can connect to the peripheral immune system through meningeal lymphatic vessels. This subversive finding reconsiders the relationship between the brain immune microenvironment and neurological diseases (Louveau et al., 2016). Immunotargeted therapy is a hot topic in glioma treatment (Agarwal et al., 2011). Glioma stem cells (GSCs) play an important role in initiating glioma, promoting tumor progression and leading to tumor recurrence (Singh et al., 2004; Sundar et al., 2014). In the microenvironment of glioma, GSCs can not only cause reprogramming of related cells in the microenvironment, but also have a high inhibitory effect on the antitumor activity of immune cells (Laterra et al., 2021). The study of GSCs in glioma immune microenvironment and targeting GSCs therapy have become the forefront focus of neurooncology. In this paper, we will review and analyze the research progress of glioma immune microenvironment and targeted immunotherapy for GSCs.

## Immune Microenvironment in Glioma

The microenvironment of glioma tissue is composed of tumor cells, immune cells and various cytokines secreted by them. These cytokines, including pro-inflammatory factors, anti-inflammatory factors and chemokines, constitute a microenvironmental network, which interact with each other to jointly regulate the regional immune effect and ultimately determine the outcome of the disease (Grabowski et al., 2021).

Glioma highly inhibits tumor immunity, and glioma cells produce immunosuppressive factors such as TGF-*β*, IL-10 and indoleamine–pyrrole 2, 3-dioxygenase (IDO) (Tran et al., 2007; Preusser et al., 2015) Glioma also expresses immune checkpoint ligand to inhibit immune response (Garber et al., 2016; Hodges et al., 2017), so it is difficult to use immunotherapy to treat glioma. In addition, Glioma tumor microenvironment is filled with a large number of regulatory T cells (Tregs), M2 tumor-associated macrophages (TAMs), and myeloid inhibitory cells (MDSCs), which are closely related to the poor prognosis of glioma (Yue et al., 2014). Many clinical trials have been conducted to suppress immune checkpoints by reinvigorating the body’s immune system against glioma (Preusser et al., 2015).

### Immune Cells

#### Macrophage and Microglia

Macrophages infiltrated by gliomas mainly come from monocytes in peripheral blood circulation, which are recruited to tumor regions and differentiated into tumor infiltrating macrophages (Sica and Bronte, 2007). Gliomas associated macrophages are usually immunosuppressive (Grabowski et al., 2021). Promote the fine direct contact between macrophages and tumors to create conditions for tumor cells to induce immunosuppression of macrophages (Zheng et al., 2013). Antitumor drugs targeting macrophages (Yu et al., 2021), such as colony-stimulating factor-1 receptor (CSF-1R), have been used to create glioma models in mice (Pyonteck et al., 2013; Sun et al., 2019; Akkari et al., 2020) found that targeting these Gliomas associated macrophages populations using a colony-stimulating factor-1 receptor (CSF-1R) inhibitor combined with radiotherapy substantially enhanced survival in preclinical models.

Microglia are both CNS-resident myeloid cells and the most important form of defense in the central nervous system. Ye et al. found that a large number of microglial cell infiltrates in gliomas, and the degree of infiltration is positively correlated with the degree of malignancy of gliomas (Ye et al., 2012). Glioma-derived factors can enhance the migration and continuous proliferation of microglia and promote the local aggregation of microglia in tumors by enhancing the adhesion kinase and PI3K/Akt pathway (Ellert-Miklaszewska et al., 2013). Microglia not only express CSF1R, but also GABA receptors (Beppu et al., 2013; Liu et al., 2016). Glutamate induces targeted chemotaxis of microglias to glioma margins and releases ligands in an AMPAR-dependent manner, promoting invasion of glioma cells and stemness activation of GSCs (Guo et al., 2009). In synaptic studies (Parkhurst et al., 2013; So-Hee et al., 2013; Miyamoto et al., 2016), microglia have been found to mediate synaptic formation through direct contact or secretion of soluble proteins BDNF or IL-10. However, GSCs induce IL-10 secretion through microglia (Wu et al., 2010), suggesting that GSCs secrete IL-10 through a bidirectional signaling axis by microglia, leading to abnormal synaptic formation, which also provides potential research direction for immunotherapy targeting GSCs.

Macrophages and microglias showed mutual activation and counterbalance in glioma immune microenvironment and tumor progression. The specific and unique roles of microglia and macrophages depend on their localization in and around the tumor (Radin and Tsirka, 2020). Studies on IDH mutated gliomas have found that macrophage transcription programs on microglias increase with tumor grade (Venteicher et al., 2017). Studies in mouse glioma models have found that microglias are predominantly located in the margins of gliomas, where they may promote spatially related behaviors such as invasion, proliferation, and stemness (Hambardzumyan et al., 2015; Shen et al., 2016; Caponegro et al., 2020). Caponegro et al. found that microglias promote the recruitment of anti-inflammatory macrophages and T regulatory cells from systemic circulation by releasing chemokines such as CCL2 (Caponegro et al., 2020). Their study also found that the enrichment of associated microglias around gliomas was associated with reduced patient survival. Microglia can also promote stem cell differentiation by releasing IL-6 (Wang et al., 2009; Zhang et al., 2012), which in turn induces GSCs to recruit anti-inflammatory macrophages by releasing osteoperioprotein (Zhou et al., 2015). An increased proportion of macrophages was found in studies of recurrent gliomas, which may indicate that macrophages play a potential role in mediating the recurrence of gliomas after radiation (Akkari et al., 2020).

#### Regulatory T Cells and Myeloid-Derived Suppressor Cells

Regulatory T cells (Treg) and Myeloid-derived suppressor cells (MDSC) play an immunosuppressive role in glioma and are important reasons for mediating immune escape. Treg does not exist in normal human brain tissue, but there are a large number of immunosuppressive Treg cells in GBM microenvironment, and the degree of Treg infiltration in glioma is closely related to tumor origin and pathological grade (Heimberger et al., 2015). Treg not only inhibits the activation of effector T cells by presenting surface antigens (Mccoy and Gros, 1999), but also inhibits the functions of other immune cells by secreting cytokines (IL-10, TGF-*β* and so on) and induces the transformation of T cells into Treg (Câmara et al., 2003; Cantini et al., 2011). Liu et al. found that Treg infiltrated densely in gliomas induced the expression of stemness related genes CD133, SOX2, NESTIN and so on, activated GSCs and promoted tumor growth by secreting TGF-*β* mediated NF-κB-IL6-STAT3 signaling pathway (Liu et al., 2021). In addition, their results showed that blocking the IL-6 receptor with tocilizumab eliminated this effect in *in vitro* and *in vivo* models.

MDSC are a type of heterogeneous cells, including immature macrophages, granulocytes, dendritic cell (DC), and other myeloid cells in the early stage of differentiation. Its main functions include promoting the increased expression of immunosuppressive molecules (IL-10, TGF-*β* and so on), inhibiting DC differentiation, inhibiting phagocytosis, reducing NK cell cytotoxicity and inducing T cell apoptosis (Rodrigues et al., 2010). Clinical studies have shown that COX-2 inhibitors (such as aspirin) can inhibit gliomas (Fujita et al., 2011). The mechanism may be that the COX-2 pathway can promote the formation of MDSC and the microenvironment of its polymerization, inhibit the infiltration of cytotoxic T lymphocytes, and thus promote the growth of glioma. The infiltration and accumulation of MDSC and Treg in GBM drives the local immunosuppression influenced by other immune cells. Chang et al. found that macrophage and microglia in the glioma microenvironment produce chemokine CCL2, which is crucial for recruiting Treg and MDSC (Chang et al., 2016). Their results showed that in a mouse glioma model, tumors growing in CCL2-deficient mice did not maximize the accumulation of Treg and MDSC.

#### Natural Killer Cells

Natural killer (NK) cells can secrete perforin and granase to induce apoptosis or necrosis of target cells. In gliomas, NK cells are the first to be recruited to the glioma region, but their function is inhibited, and the degree of functional inhibition is positively correlated with the degree of malignancy of gliomas (Dix et al., 1999). Glioma cells can inhibit NK cells’ function not only by direct contact, but also by secreting inhibitory factors (Aldemir et al., 2005; Hao et al., 2011; Wolpert et al., 2012). In addition, (Orozco-Morales et al., 2015) found that the specific changes of tumor suppressor factor (Rb) and proto-oncogene (Ras) were also an important reason for the resistance of glioma cells to NK cell-mediated cytotoxicity. Therefore, adoptive immunotherapy based on NK cells is a promising immunotherapeutic strategy for glioma. *In vitro* studies have shown that GSCs can express high levels of PVR and adhesin 2, and NK cells activated receptor DNAM⁃1 can specifically recognize the above two ligands, thus killing GSCs (Castriconi et al., 2009). They also found that GSCs derived from GBM were highly sensitive to NK cell-mediated live lysates stimulated by IL⁃2 or IL⁃15 (Castriconi et al., 2009).

### Cytokines

In the tumor microenvironment, cytokines are communication mediators between cells and tissues, and are closely related to the differentiation and activation of immune cells in the tumor microenvironment. Immunoregulatory cytokines such as pro-inflammatory factors (IL-1, IL-2, TNF-α, IFN-*γ*), anti-inflammatory factors (IL-10, TGF-*β*), and chemokines (CCL-2, CCL-5, CCL-7, CX3CL1) are synthesized and secreted in the microenvironment of glioma. These cytokines and their receptors form a comprehensive regulatory network in the local tumor, and play an important role in tumor progression and tumor immune response. Here we introduce some of the latest and most popular factors.

#### Transforming Growth Factor⁃β

Transforming growth factor⁃β (TGF-*β*) signal transducing pathway is involved in multiple links of glioma genesis and malignant progression, and the change of TGF-*β* expression in serum of patients with malignant glioma is positively correlated with tumor grade and prognosis. TGF-*β* can induce monocyte recruitment, macrophage phagocytosis inhibition, tumor local T cell proliferation inhibition and other effects (Wu et al., 2010). TGF-*β* signal transduction pathway mainly regulates stem cell-related genes (such as SOX2 and SOX4) to achieve self-renewal and inhibit differentiation of GSCs (Ikushima et al., 2009; Peñuelas et al., 2009). Activation receptor NKG2D expressed by NK cells and CD8^+^T cells plays a role in specific killing of transformed cells. Crane et al. found that GBM could down-regulate NKG2D by secreting TGF-*β*, thus inhibiting the killing function of NK cells and CD8^+^ T cells (Crane et al., 2010).

#### Chemokines

Chemokines play an important role in the local migration of immune cells such as microglias and macrophages to tumors. Studies have shown that the high expression of inflammatory chemokines ligand 1 (CXCL1) and ligand 2 (CXCL2) is closely related to tumor invasion, metastasis and poor prognosis (Miyake et al., 2014; Seifert et al., 2016). CXCR2 is the only receptor for CXCL1 and CXCL2, and is highly expressed mainly in myeloid cells, including neutrophils, monocytes and macrophages. These receptors guide the migration of myeloid derived cells from the bone marrow to tumor regions where CXCL1 and CXCL2 are highly expressed, inhibit the activity and proliferation of effector T cells, and stimulate the growth of regulatory T cells, thereby promoting tumor immune escape (Oppenheim et al., 1991; Gabrilovich et al., 2012). In addition to suppressing immunity, CXCL1 and CXCL2 also recruit myeloid cells to produce paracrine factors such as S100A9 and promote tumor cell survival (Acharyya et al., 2012; Alafate et al., 2020) found that silencing CXCL1 can down-regulate NF-κB and mesenchymal cell transformation, and inhibit the growth of human glioma xenograft (Alafate et al., 2020).

Hu et al. found that the high expression of CXCL1 and CXCL2 was closely related to the invasiveness of glioma. The high expression of CXCL1/CXCL2 promoted the migration of myeloid cells and disrupted the accumulation of CD8^+^T cells in the tumor microenvironment, leading to accelerated tumor proliferation. Inhibition of CXCL1/2 significantly prevented myeloid inhibitory cell migration and increased CD8^+^T cell accumulation *in vitro* and *in vivo*. CXCL1/2 also promoted the expression of paracrine factor S100A9, activated ERK1/2 and p70S60k, and promoted tumor growth, while blocking CXCL1/2 down-regulated the expression of these pro-survival factors and slowed tumor growth. Targeting CXCL1/2 with standard chemotherapy can improve the chemotherapy efficiency of glioma and prolong the survival of glioma mice (Hu et al., 2021).

#### Oncostatin M

Oncostatin M (OSM) is another cytokine with important biological significance. Its main function is to inhibit the growth of various tumor cells and induce the differentiation of some tumor cells. Oncostatin M receptor (OSMR) is widely distributed on the surface of many tumor cells, endothelial cells and epithelial cells. The inhibitory effect of tumor cell growth and differentiation induced by statin M is exerted by specific high-affinity receptors (Auguste et al., 1997; Liu et al., 1998). Previous single-cell RNA sequencing by researchers found that malignant glioma cells had four states: neural progenitor cell-like (NPC-like), oligodendrocyte progenitor cell-like (OPC-like), astrocyte like (AC-like) and mesenchymal like (MES-like), indicating that tumor cells have potential state plasticity (Tanay and Regev, 2017; Neftel et al., 2019). The frequency of the first three states in tumors is affected by some driving genes (such as CDK4, PDGFRA, EGFR, etc.). Mesenchymal tumor cells are almost nonexistent in normal human brain tissue and are associated only with genetic changes, and they have been poorly studied. Previous studies have shown that TCGA-MES subtype is related to the abundance of macrophages, and NF1 mutation or deletion in TCGA -MES rich tumors increases the recruitment of macrophages (Wang et al., 2017). Therefore, TCGA-MES is related to the increased abundance of macrophages in tumors, which may explain its advantage in recurrent diseases (Qzawa et al., 2014; Hara et al., 2021) found that MES status of GBM was closely related to macrophage expression, T cell abundance, and increased cytotoxicity in both the mouse model and GBM globule model. The results of this study show that OSM secreted on macrophages induces the transition of MES like glioma cells to MES like state. In addition, OSM-mediated induction mainly comes from the regulation of STAT3, and MES-like tumor cells are also associated with increased mesenchymal expression of macrophages and increased cytotoxicity of T cells. This study highlights the influence of changes in immune microenvironment on tumor cell state, and the combination of changes in tumor cell state and activation of the immune system has a good potential therapeutic effect.

The local aggregation of the above immune cell subsets and cytokines in glioma fully demonstrates that they jointly form an immunosuppressive microenvironment in glioma and hinder the production of anti-tumor immune response. Therefore, how to reduce or reverse this state of inhibition, will continue to receive attention.

## GSCS and Glioma Immune Microenvironment

Tumor stem cells account for a small proportion of tumor cells, and have the biological characteristics of self-renewal, multilineage differentiation and tolerance to conventional therapy. GSCs are the cells that play a tumor initiation role in glioma (Sundar et al., 2014). With high heterogeneity, they can induce tumor angiogenesis, promote tumor invasion and dissemination, and are highly tolerant to radiotherapy and chemotherapy (Folkins et al., 2007). Under the pressure of conventional treatment, they can rapidly rebuild tumors, leading to rapid recurrence of glioma (Zhi et al., 2010). The surface of GSCs can express different proteins, which are closely related to the maintenance of their homeostasis. These cell surface proteins are ideal markers for screening and targeting GSCs, among which CD133 is the most classic (Sundar et al., 2014). Other well-studied surface markers include SOX2, KLF4, C-myC, Nestin, Oct4 and A2B5, etc. Early studies targeted GSCs by preparing specific monoclonal antibodies targeting these markers. Cells in the GSCs microenvironment can secret a variety of cytokines, such as vascular endothelial growth factor (VEGF), hypoxia-inducing factor (HIF), and fibroblast growth factor 2 (FGF2), to stimulate GSCs to self-renew, induce angiogenesis, recruit immune cells, and promote tumor cell invasion and metastasis (Hambardzumyan and Bergers, 2015).

### Immunotherapy Targeting GSCs

#### Oncolytic Virus Targeting GSCs

Oncolytic viruses (OVs) are therapeutics designed to selectively multiply and kill tumor cells. An important design principle is to weaken or delete virulence factors so that the oncolytic virus cannot replicate in normal tissues, but still retain the ability to replicate and kill tumor cells in tumor cells, at the same time it also can stimulate the immune response, attract more immune cells to continue to kill residual cancer cells (Muik et al., 2014; Fukuhara et al., 2016). However, the ability of oncolytic viruses to spread, cross the host tumor and penetrate the blood-brain barrier is very limited, which limits their application in glioma. Neural Stem Cells (NSCs) are pluripotent progenitor Cells derived from the developing and adult central nervous system (Conti and Cattaneo, 2010). Preclinical experiments have shown that NSC can cross the blood-brain barrier, reach the tumor region, surround the tumor boundary, and migrate within the brain to target glioma cells (Aboody et al., 2000). The ability of NSCs to migrate and spread freely within tumors can be used to deliver therapeutic molecules across the blood-brain barrier to target glioma cells. Kim et al. modified an oncolytic adenovirus (CRAd-S-pk7), which improved viral replication and targeting to glioma cells, and increased the anti-tumor activity and survival rate of mice (Kim et al., 2016). By combining the tumor propensity of NSCs with the ability of CRAd-S-pk7 to target GSCs, they engineered an NSC-provided engineered oncolytic virus (NSC-CRAd-S-pk7), which extended the median survival of mice treated with it by 50%. In a recently published phase one trial, NSC-CRAd-S-pk7 injection was shown to be safe and effective in patients with newly diagnosed GBM during surgery (Fares et al., 2021). The results also suggest that multi-site injection in the brain can increase virus coverage and improve treatment efficiency, and that concurrent use of chemoradiotherapy can enhance the replication capacity of oncolytic adenovirus, which is expected to further improve treatment effectiveness. Neurotropic viruses as oncolytic viruses in the treatment of brain tumors have become the focus of attention because of their ability to cross the blood-brain barrier. The Zika virus (ZIKV) outbreak in 2015 became a global public health emergency. This latest study shows that ZIKA virus can target glioblastoma stem cells through the SOX2-integrin *α*V*β*5 axis to exert antitumor effect (Zhu et al., 2020). They found that ZIKV was more likely to infect GSCs from the patient’s source. At the same time, ZIKV could significantly induce apoptosis and inhibit proliferation of GSCs. SOX2 is an important regulator of GSCs with high expression, which can induce cell pluripotency and maintain the characteristics of stem cells. After knocking down SOX2, GSCs are less susceptible to ZIKV infection. Integrin *α*V*β*5 was associated with the expression of SOX2 and other GSC markers. Inhibition of *α*V*β*5 significantly reduced viral infection and inhibited the effect of ZIKV on neuroglobulogenesis. Integrin αVβ5 plays an important role in ZIKV infection and cell damage. Therefore, the results of this study confirmed the oncolytic activity of ZIKV against GSCs and correlated with *α*V*β*5 expression. Friedman et al. found that the modified HSV-1 oncolytic virus (G207) is highly sensitive to treating high-grade gliomas in children. The results showed that G207 could transform immunologically “cold” tumors into “hot” tumors (Friedman et al., 2021). In addition, because the GSCs are highly enriched in integrins such as *α*V*β*3 or *α*V*β*5, the oncolytic viruses designed by genetic engineering technology can enter the glioma stem cells through these integrins and play an anti-tumor role, of which DNX⁃2401 is an oncolytic virus with tumor specificity and strong infectivity. It has been used in phase I clinical trials with good safety, significantly reduced tumor volume and prolonged patient survival (Jiang et al., 2007; Lang et al., 2018).

#### Tumor Vaccines Targeting GSCs

Tumor vaccines can activate the host immune system to recognize and kill tumor cells, which is an active immunotherapy. The best way to activate the immune system is to stimulate multifunctional antigen presenting cells (APC), such as DC, macrophages, and B lymphocytes (B cells), among which DC is the best choice. GSCs have the same ability to sensitize DC, so the direct targeting of this tumor cell subpopulation and the establishment of tumor vaccines against glioma are expected to be effective therapeutic programs (Finocchiaro and Pellegatta, 2015). In recent years, SOX2, as an important transcription factor for maintaining glioma stem cells, has been considered as a new target for active immunotherapy. SOX2 peptide vaccine can significantly enhance systemic and local immune responses, and prolong the survival of tumor-bearing animals in tumor models, with definite efficacy observed regardless of whether combined with chemotherapy (Favaro et al., 2014). Therefore, glioma stem cell-related vaccines targeting SOX2 and other glioma stem cell-related genes may provide a new regimen for active immunotherapy. Epidermal growth factor receptor variant III (EGFRvIII), the most common EGFR gene specific mutation in glioma and GSCs. In recent glioma studies, it has been found that the EGFRvIII resulting from the in-frame deletion of the EGF receptor gene is not only overexpressed and carcinogenic, but also exists in tumor stem cells with high immunogenicity. Therefore, it has great potential as a target for immunotherapy. As a synthetic peptide that can specifically recognize EGFRvIII, pepVIII has shown a good application prospect in the test of glioma. In particular, in the latest glioma study, a variant peptide vaccine (Y6-pepVIII) designed by the researchers was found to significantly enhance the immune response and improve survival in mice (Fidanza et al., 2021). Their results showed an increased proportion of CD8^+^ T cells in tumors of mice receiving Y6-pepVIII vaccine, and both CD4^+^ and CD8^+^ T subsets were involved in antitumor responses.

#### Monoclonal Antibodies Targeting GSCs

Monoclonal antibodies (Mabs) play an important role in tumor immunotherapy due to their direct cell-killing and immunomodulatory effects (Guo et al., 2011). Previous clinical studies on anti-EGFR antibody in glioma have found that antibody-mediated therapy has a potential application prospect (Herbst and Shin, 2002). However, bevacizumab, the first monoclonal antibody against endothelial production factor (VEGF) approved by the US Food and Drug Administration (FDA) for the treatment of high-grade gliomas, has significant anti-tumor angiogenesis. It will greatly improve the killing and inhibition of glioma stem cells by other therapies (Calabrese et al., 2007), but interestingly, no significant survival benefit has been observed in subsequent clinical trials (Zhuang et al., 2016). Recent studies have found that Semaphorin3A (SEMA3A), as an axon guide factor, has carcinogenic effect in several cancers (Kigel et al., 2008). Lee et al. found that SEMA3A was highly expressed in human glioma specimens. The SEMA3A antibody developed by them significantly inhibited the migration and proliferation of GBM patient-derived cells and U87-MG cells *in vitro*, and inhibited tumor by down-regulating cell proliferation dynamics and tumor-associated macrophage recruitment in PDX model (Lee et al., 2018). These different findings need to be further explored.

### Therapeutic Strategies for Immunosuppression of GSCs Microenvironment

Recent studies have shown that there is a mechanism in the immune microenvironment of tumor stem cells that inhibits the immune response and interferes with the surveillance role of the immune system. Immune checkpoints play a key role in the immunosuppressed tumor stem cell microenvironment, and the intervention of these immune checkpoints has become an important target of tumor immunotherapy (Pardoll and Drew, 2012; Topalian et al., 2015). During tumorigenesis, the immune checkpoint interaction between GSCs and immune cells changes from stimulating to inhibiting (Zhai et al., 2020).

The most representative immune checkpoints are cytotoxic T lymphocyte-associated protein antigen-4 (CTLA-4) and programmed cell death-1 (PD-1) (Buchbinder and Desai, 2016). CTLA-4 is a negative regulator of T cells and has a high affinity with T cells, which leads to depletion and inhibits their activation (Four et al., 2016; Chikuma, 2017). Anti-CTLA-4 treatment can remove the inhibitory effect of T cells, thus supporting the anti-tumor immune response (Fecci et al., 2014). PD-1 is expressed in various immune cells (Alsaab et al., 2017) and is highly expressed in malignant tumor tissues, which is associated with T cell depletion (Ohaegbulam et al., 2015). The interaction between CTLA-4 and PD-1 significantly inhibited the secretion of IFN⁃*γ* by activated T cells and impaired the function of T cells (Que et al., 2017). Therefore, joint blocking of CTLA-4 and PD-1 signal transduction pathway may be an effective way to rescue immunosuppression of malignant tumors including glioma and maintain anti-tumor immune response (Castro et al., 2014). Microrna (miRNA) regulate multiple gene transcripts and may involve multiple immune checkpoints, so they have potential as immunotherapies. Wei et al. found that mir-138 could bind the 3′ untranslated regions of CTLA-4 and PD-1, and mir-138 transfected human CD4^+^ T cells could inhibit the expression of CTLA-4 and PD-1 in transfected human CD4^+^ T cells (Wei et al., 2016). Immune checkpoint inhibitors that block the molecule CTLA-4 (2011) and PD-1 (2014) have been approved by the FDA. Among the immunotherapy drugs against PD-1 antibody, Nivolumab has attracted the most attention. In clinical efficacy observation, it was found that combined radiotherapy, chemotherapy and electric field therapy can improve the survival of some glioma patients to a certain extent (Preusser et al., 2015), improve prognosis and reduce adverse drug reactions (Chandran et al., 2017).

In addition, GSCs reduce T cell cognition and evade systemic immune monitoring by downregulation or defect of major histocompatibility complex I (MHC-I) molecules and antigen-processing mechanism (APM) components. Improving tumor surface antigens of GSCs may be an effective strategy for triggering adaptive immune responses and activating cytotoxic T cells (CTLs) to inhibit gliomas. A new research found that inhibition of histone deacetylase (HDAC) increased the expression of MHC-I and APM components, and enhanced the antitumor effect of tumor lysate vaccine by activating the destruction of CTLs (Yang et al., 2020). The enhanced T cell immune response induced by HDAC inhibition may provide a new direction for targeting GSCs-based immunotherapy.

## Conclusion

**FIGURE 1:**
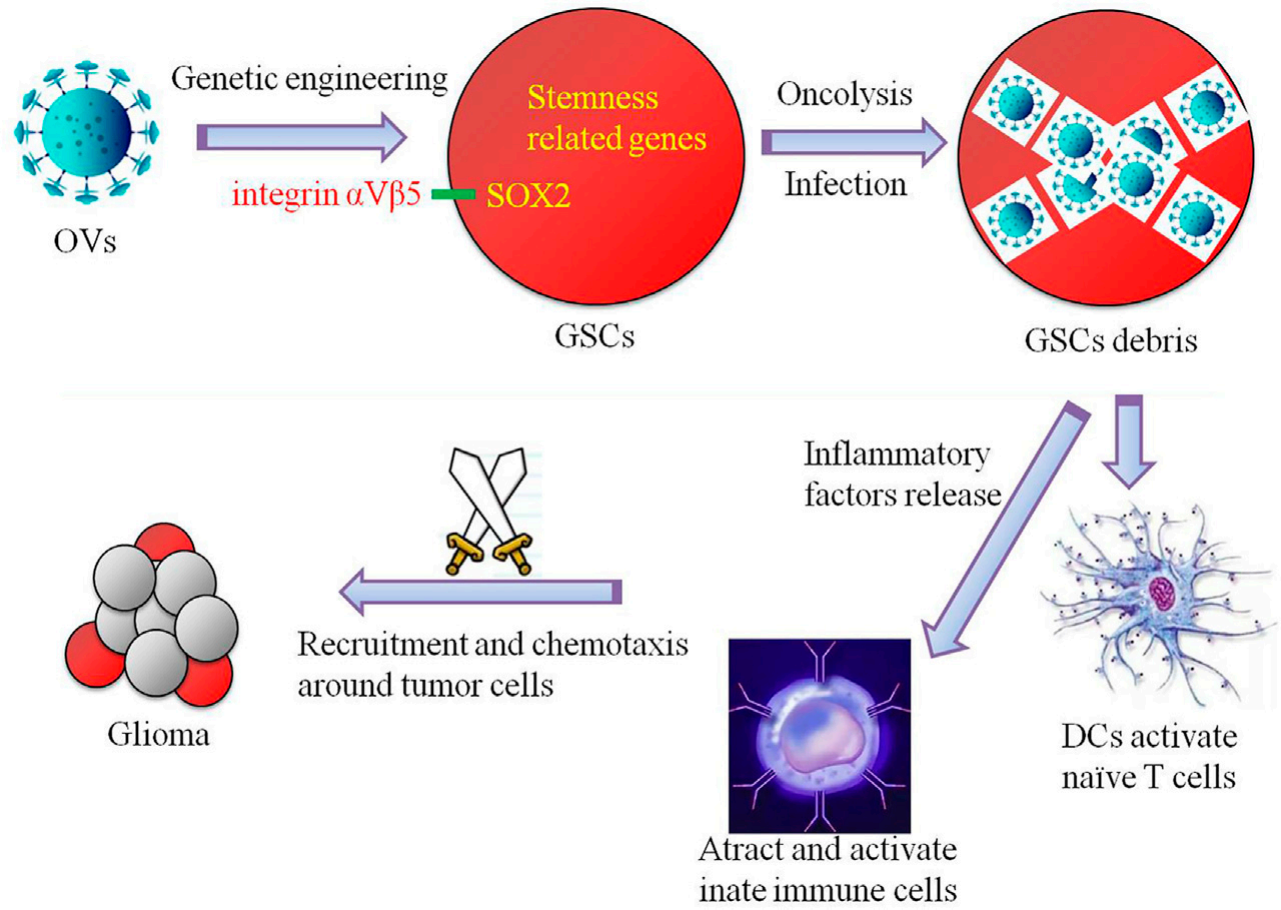
Schematic diagram of a simple simulation of the mechanism by which oncolytic viruses (OVs) targeting glioma stem cells (GSCs) exert glioma-inhibiting action. GSCs infected by genetically engineered oncolytic viruses that target dry-associated genes (e.g., SOX2-integrin *α*V*β*5 axis) can be cleaved directly to destroy GSCs, and additional immune cells are recruited and chemotaxis around gliomas through the release of inflammatory factors to kill and inhibit gliomas.

GSCs and glioma immune microenvironment are the key factors in the development and progression of glioma. The complex multidirectional interactions between GSCs, various immune cells and cytokines eventually form a tumor-supporting immune microenvironment that promotes tumorigenesis, proliferation and invasion initiated by GSCs. The study on the immunosuppression mechanism of GSCs and glioma microenvironment will help us to further explore the immunotherapy strategies targeting GSCs and point out new directions for establishing effective therapeutic targets. We always believe that immunotherapy is the “ultimate killer” of glioma. Oncolytic virus immunotherapy targeting GSCs is the blade of this “sharp weapon” (Figure 1). In the context of the global COVID-19 pandemic, while we conquer it, its research in tumor and even glioma is worth paying close attention and thinking.
